# High-accuracy detection of early Parkinson's Disease using multiple characteristics of finger movement while typing

**DOI:** 10.1371/journal.pone.0188226

**Published:** 2017-11-30

**Authors:** Warwick R. Adams

**Affiliations:** School of Computing & Mathematics, Charles Sturt University, N.S.W., Australia; University of Bonn, Bonn-Aachen International Center for IT, GERMANY

## Abstract

Parkinson’s Disease (PD) is a progressive neurodegenerative movement disease affecting over 6 million people worldwide. Loss of dopamine-producing neurons results in a range of both motor and non-motor symptoms, however there is currently no definitive test for PD by non-specialist clinicians, especially in the early disease stages where the symptoms may be subtle and poorly characterised. This results in a high misdiagnosis rate (up to 25% by non-specialists) and people can have the disease for many years before diagnosis. There is a need for a more accurate, objective means of early detection, ideally one which can be used by individuals in their home setting. In this investigation, keystroke timing information from 103 subjects (comprising 32 with mild PD severity and the remainder non-PD controls) was captured as they typed on a computer keyboard over an extended period and showed that PD affects various characteristics of hand and finger movement and that these can be detected. A novel methodology was used to classify the subjects’ disease status, by utilising a combination of many keystroke features which were analysed by an ensemble of machine learning classification models. When applied to two separate participant groups, this approach was able to successfully discriminate between early-PD subjects and controls with 96% sensitivity, 97% specificity and an AUC of 0.98. The technique does not require any specialised equipment or medical supervision, and does not rely on the experience and skill of the practitioner. Regarding more general application, it currently does not incorporate a second cardinal disease symptom, so may not differentiate PD from similar movement-related disorders.

## Introduction

### The research problem

Parkinson’s Disease (PD) is a progressive neurodegenerative movement disease affecting approximately 2% of people at the age of 65 and is the most second most commonly occurring neurodegenerative disease in the elderly (after Alzheimer’s Disease), with more than 6.3 million people worldwide with PD [1]. In PD sufferers, loss of dopamine-producing neurons results in a range of both motor and non-motor symptoms and currently there is no cure, no means of slowing the disease progression, and no means of prevention. From the perspective of patient quality of life, PD is one of the most severe of all chronic diseases.

At present, diagnosis relies on observation of a combination of visible symptoms by a specialist (typically a neurologist), however PD is commonly either misdiagnosed or the diagnosis is missed completely. Pagan [2] found that, based on a UK autopsy study, there was a misdiagnosis rate of 24% and that this strongly depended on who was performing the diagnosis and whether or not they were applying diagnostic criteria based on clinical guidelines. Specialists who were not movement disorder experts had a correct diagnosis rate of only 75% and diagnoses by primary care doctors had a correct diagnosis of just 53%. In contrast, movement disorder specialists were mistaken by only 6% to 8%, which raises an obvious issue–in order to be referred on to a movement specialist, the patient’s primary health practitioner must first recognise and diagnose the symptoms. In addition, a patient may have the disease for 5 to 10 years before it is diagnosed [3] and, by the time of diagnosis, typically 70% of the neurons in the affected part of the brain (the substantia nigra) have already been lost [4].

With regard to disease diagnoses more generally, there are various physical and functional biomarkers which can be used to provide both diagnostic and predictive information (for example in predicting responses to therapies and drugs). Human–computer interaction (HCI) researches the interfaces between people and computers, producing markers that can be used to measure the state of the user, for example, physiological, cognitive and mental states [5]. In principle, any device which users interact with, and which produces an output that can be measured and stored, could be utilised as part of HCI—devices such as computers, smartphones, tablet computers, gaming platforms and wearable devices. However, technology-based assessments must also provide valid and accurate results, be independent of rater's training, and allow easy and repetitive use [6].

PD results in a range of both motor and non-motor symptoms, with the effects on movement including slowness, sidedness, jerkiness and tremors. The hypothesis of this research study was that PD could be detected in its early stages in a person by changes in the characteristics of finger movement as they typed on a keyboard, and that such changes could be used to distinguish and classify people with PD from those without the disease.

There have been previous HCI studies into the diagnosis of PD using features of movement and gait, speech analysis, gripping and lifting tasks, finger tapping tests, hand and finger movement, and handwriting. To date, such studies have all shown limitations in one or more facets—in the sensitivity and specificity of results, the level of specialist supervision or intervention needed, or the requirement for specialised equipment—which have prevented their application more generally as tools to detect or diagnose PD.

### Significance of the research

Generally, by the time of diagnosis of PD, the disease is already well advanced, significant neuron loss and damage has already occurred, and any possibility of delaying further disease progression or providing neuroprotection is unlikely. The goal must be to diagnose and treat PD well before the irreversible destructive changes have taken place [2], ideally at least 5 years earlier than is currently the case. In addition, because the most severe symptoms occur in the advanced stages of the disease, strategies aimed at early detection and treatment will have the most benefit [7].

The objective of this research was to identify those characteristics of finger movement which are affected by PD and, through the application of machine learning (ML), to be able to accurately classify the disease status of the participants in the investigation.

### Symptoms of Parkinson’s Disease

The cardinal features of PD are tremor, bradykinesia, postural instability, muscle rigidity and motor blocks [8], however there are also a wide range of other motor and non-motor symptoms.

Rest tremor is the most common and easily recognised symptom of PD, present in 70% to 75% of cases. The tremors occur at a frequency of 4 to 6 Hz and are prominent at the distal part of an extremity such as the hands [8] and can also involve lips, chin, jaw and legs. Rest tremors typically disappear with action and during sleep.

Bradykinesia is characterised by a slowness of initiating voluntary movement and in sustaining repetitive movements with progressive reduction in speed and amplitude [9] and is the most characteristic feature of PD. Bradykinesia is symptomatic of all basal ganglia disorders [8] and is typified by difficulty with performing sequential and simultaneous tasks. According to Jahanshahi et al. [10] the initial manifestation of PD is often slowness in performing the normal activities of daily life, especially those tasks requiring fine motor control.

Particularly in its early stages, PD is characterised by a predominantly unilateral (asymmetrical) appearance of the motor symptoms [11]. This sidedness can be so conspicuous that it often serves as a clinical parameter to differentiate the disease from other neurodegenerative Parkinsonian syndromes, for example, in multiple system atrophy, diffuse Lewy body disease and progressive supra-nuclear palsy, there is usually no side predominance [12].

Rigidity in PD sufferers is characterised by increased resistance, present throughout the range of movement of a limb. When accompanied by an underlying tremor it results in a cogwheel phenomenon, which continues throughout an entire range of movement. The rigidity may occur at the neck, shoulders or hips (proximally) or wrists and ankles (distally) [8].

### Issues with early diagnosis

The problems with diagnosing PD arise because there is no definitive test, and currently the disease diagnosis must be based on clinical and observational criteria only. Many of the symptoms of PD are imprecise and also common to other diseases, both neurodegenerative and non-neurodegenerative in nature. Evaluation may be performed using the Unified Parkinson’s Disease Rating Scale (UPDRS) [13], a tool based on a score derived from the neurological evaluation that is performed by a physician, and hence it is a subjective measure which leads to a lack of objectivity, repeatability and sensitivity in the scale [14].

Parkinson's disease is usually preceded by a premotor phase that can last for years, or even decades, between the onset of neurodegeneration and manifestation of the classic clinical motor symptoms. The most common pre-diagnostic symptom of Parkinson’s Disease within 2 years before diagnosis is tremor, with 41% of individuals reporting symptoms to their medical practitioner compared with less than 1% of controls, and the incidence of tremor is already higher at 5 and 10 years before diagnosis [3]. Despite the reliance on motor symptoms for the standard diagnosis of PD, premotor symptoms hold promise for the early diagnosis of PD and considerable progress has been made in recent years in establishing premotor symptoms as a means of identifying PD much earlier [2]. Biomarkers hold promise for reliable early PD diagnosis, while neuroimaging and sonography show enormous potential for high degrees of sensitivity and specificity in diagnosing early PD [2].

### Biometrics and keystroke dynamics

The use of keystrokes as a means of identification has a long history. Das et al. [15] used keystroke dynamics while typing a computer login string to identify users with 90% to 99% accuracy. Their technique involved key hold times and latency, using a Gaussian mixture model and a neural network. This, and many other similar studies [16,17,18,19], demonstrate that keystroke characteristics can be used very accurately to classify the features of particular users.

Keystroke dynamic features can be extracted using the timing information of the key press and release events. The hold time of individual keys and the latency between keys (the time interval between pressing one key and a succeeding key) are typically exploited [20]. In addition to ordered pairs (two successive keystrokes), n-tuples of a sequence of keystrokes have also been investigated and keystroke biometrics research has utilised many machine learning and classification techniques.

### Existing studies into Parkinson’s biomarkers

It is known that many PD biomarkers can be analysed using various forms of human-computer interaction, including movement and gait analysis, speech analysis, the precision grip and lift test (PLGT), finger tapping tests (FTT), hand and finger movement, and handwriting.

In non-PD subjects, finger tapping frequency declines with advancing age, men tap faster than women, and tapping with the dominant finger is faster than that of the non-dominant finger. The basal ganglia facilitate sequential movement and the sequence of movements, however in PD patients bradykinesia and disturbances of rhythm formation occur which can be assessed by FTT. Using an accelerometer and touch sensor, Yokoe et al. [21] measured 14 parameters of FTT movement, showing clear differences between PD and non-PD patients. By classifying these into both velocity and amplitude parameters and rhythm-related parameters, they found that maximum opening velocity was the most sensitive measure and most closely aligned with the UPDRS FTT score.

People with PD tend to have slower reaction times than non-effected people of similar age and this can be investigated with regard to the sequence of mental steps that occur between the time that a stimulus is presented and the subsequent physical response [22]. Reaction times can be separated into either a simple response (such as pressing one key) or a complex response (pressing a sequence of keys). Low et al. [22] found that, especially when complex responses are required, the reaction times of PD patients were slower, both in the delayed onset of pre-motor processes and the motor responses themselves. Another effect of PD is increasing difficulty in performing sequential and bi-manual movements [23]. Pal et al. [24] suggested that analysis of sequential hand and finger movements may provide for indication of PD, then Giancardo et al. [25], prompted by the use of keyboard typing characteristics in biometrics, utilised the typing of people on a computer keyboard as a means of observing and potentially quantifying motor impairment such as in PD. Using a time series analysis of keystroke hold times and a support vector machine (SVM) they showed significant differences between PD patients and controls, but a related follow-on study of typing characteristics with a larger group [26] only achieved an accuracy of 78%. Notably, none of these studies appeared to consider the asymmetry of movement (between left and right hands), although such sidedness is a significant feature in early PD.

A conclusion from previous studies is that PD affects multiple aspects of hand and finger movements and that many of these may be detected (both singly and in combination) through changes in the response characteristics as people type a sequence of text on a computer keyboard.

## Materials and methods

### Human-computer interaction

The existing research concerning PD diagnosis through the various motor symptoms has shown that there are a range of features present during typing on a keyboard, including–

- Reaction speed (the equivalent of a finger tapping test)
- Slowness of movement (both keystroke hold times and latency times between sequential keys), both for same-sided and opposite-sided fingers
- Indications of sidedness (asymmetry of responses between left and right hands)
- Degradation in repetitive movement and typing of sequences of letters (n-tuples)
- Variability of movement and signs of hand and finger tremors
- Jerkiness of motion (hesitations and pauses)
- Changes throughout the day (diurnal effects of tiredness)
- Changes over time as the disease progresses

It should be noted that in bradykinesia there is both a slowing of the initiation of movement (the response time, defined here as the time from the brain intending to initiate a movement until the resulting movement occurs), as well as in the speed of such movement. Even though response time cannot be directly measured from keystrokes, the differences in latency between successive keystrokes provide an insight into both the response times and any asymmetry between left and right sides (e.g. the difference between a ‘LR’ ordered pair and an ‘RL’ one).

**Fig 1:**
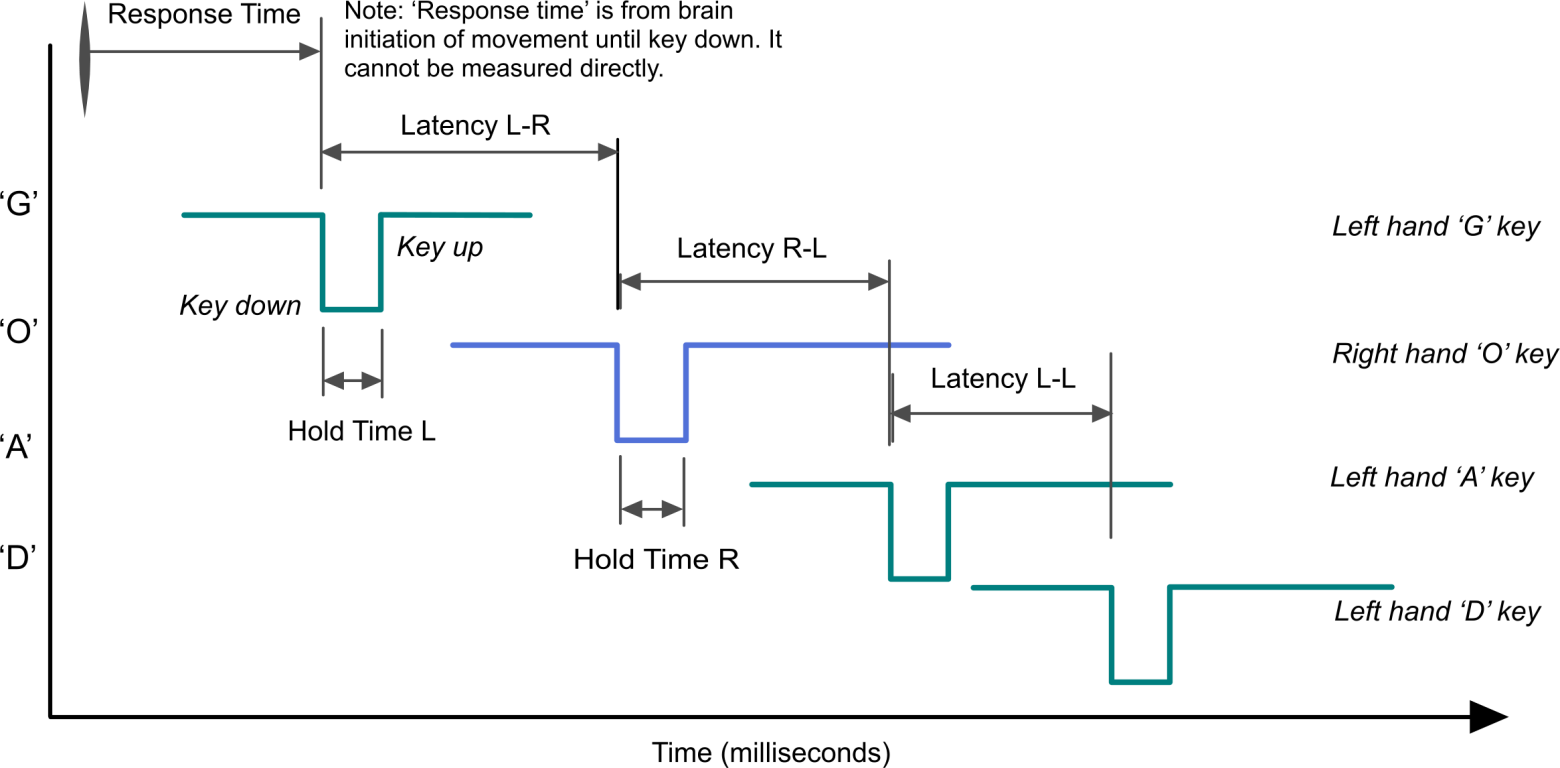
Example of keystroke timings while typing the word ‘GOAD’.

In this investigation, the keystroke dynamics of participants typing on a computer keyboard were captured as they typed normally throughout the day. This was a significant aspect of the investigation, as it meant that the participants were not limited to a directed typing task, the keystroke monitoring was completely un-intrusive upon their normal routine, and no external supervision was involved. The procedure involved each participant installing a small software application (‘Tappy’), on their Windows computer, which recorded each keypress event and its timing, along with the key’s position on the keyboard and whether it was a left or right-handed key. The specific keystroke timing events that were captured are shown in Table 1 and the manner in which these timings occur during typing the sequence of letters comprising the word ‘Goad’ is shown in Fig 1. The method involved monitoring all the participants’ typing, irrespective of the application they were using at the time (e.g. typing emails and documents), and not being limited to a ‘typing test’ of predetermined passages of text. This design decision was made in order to facilitate data capture over a longer timeframe, as well as to avoid any stress on the participant to perform well, which could itself change their keystroke dynamics.

**Table 1:** Keystroke events as successive keys are pressed and released.

Keystroke Data	Definition	Comments
Timestamp	The time of day (hh:mm:ss.sss).	The time at which each keystroke began.
Hold Time	The elapsed time (ms) between the Key Down and Key Up events when pressing and releasing a key.	Hold Time is a measure of how quickly the finger is tapped and can also indicate the relative force of tapping. Hold Times are typically in the range of 60 to 140 ms.
Latency	The elapsed time (ms) from the Key Down of one key until the Key Down of the subsequent key.	Latency between successive keystrokes can be separated into same-hand (LL and RR) and opposite-hand (LR and RL). Since the space key can be pressed by either hand, it was treated separately (LS, RS, SL, SR). Latency is typically anywhere in the range of 50 to 800 ms (anything greater than that was considered to be a pause in typing).
Flight Time	The elapsed time (ms) between releasing a key and pressing the subsequent key.	*Flight Time* = *Latency* − *Hold Time*

### Study participants

This study was approved by the Human Research Ethics Committee (HREC) at Charles Sturt University, protocol number H17013, and was conducted according to The National Health and Medical Research Council Australia requirements [27], including the informed consent of all participants and the anonymity of both participants and their data. Over the period of July 2016 to March 2017 potential research participants visited the research website, which provided details of the project, the need for volunteers and the eligibility criteria (aged between 50 and 80). Those interested in participating then downloaded and installed the keystroke capture application and entered details regarding their age and disease status (Table 2). During the software installation steps the participant was required to accept the Informed Consent before they could proceed further.

**Table 2:** Participant details which were recorded.

Data Element	Details
Parkinson’s (Y/N)	Whether or not they had already been diagnosed with PD.
When diagnosed (Year)	How long had they had the disease for.
Tremors (Y/N)	Approximately 70% of PD patients have tremors.
Sidedness (Left, Right, None)	60% of PD patients are affected more on one side than the other. This potentially correlates with asymmetry of keystroke features.
UPDRS rating (1 to 5)	The Unified Parkinson’s Disease Rating Scale (if known).
Impact (Mild, Medium, Severe)	The disease severity currently and impact on their daily life.
Medication Y/N (Levodopa/Carbidopa, MAO-B inhibitor, Dopamine agonist, Other)	Whether or not they were taking PD medication. In particular, levodopa lessens the motor symptoms and could mask the keystroke characteristics.
Age (Birth year)	Used to correlate the effects of normal aging on the characteristics (e.g. slowing of movement).
Gender (M, F)	There could be gender-specific differences.

### Data collection and processing

**Fig 2:**
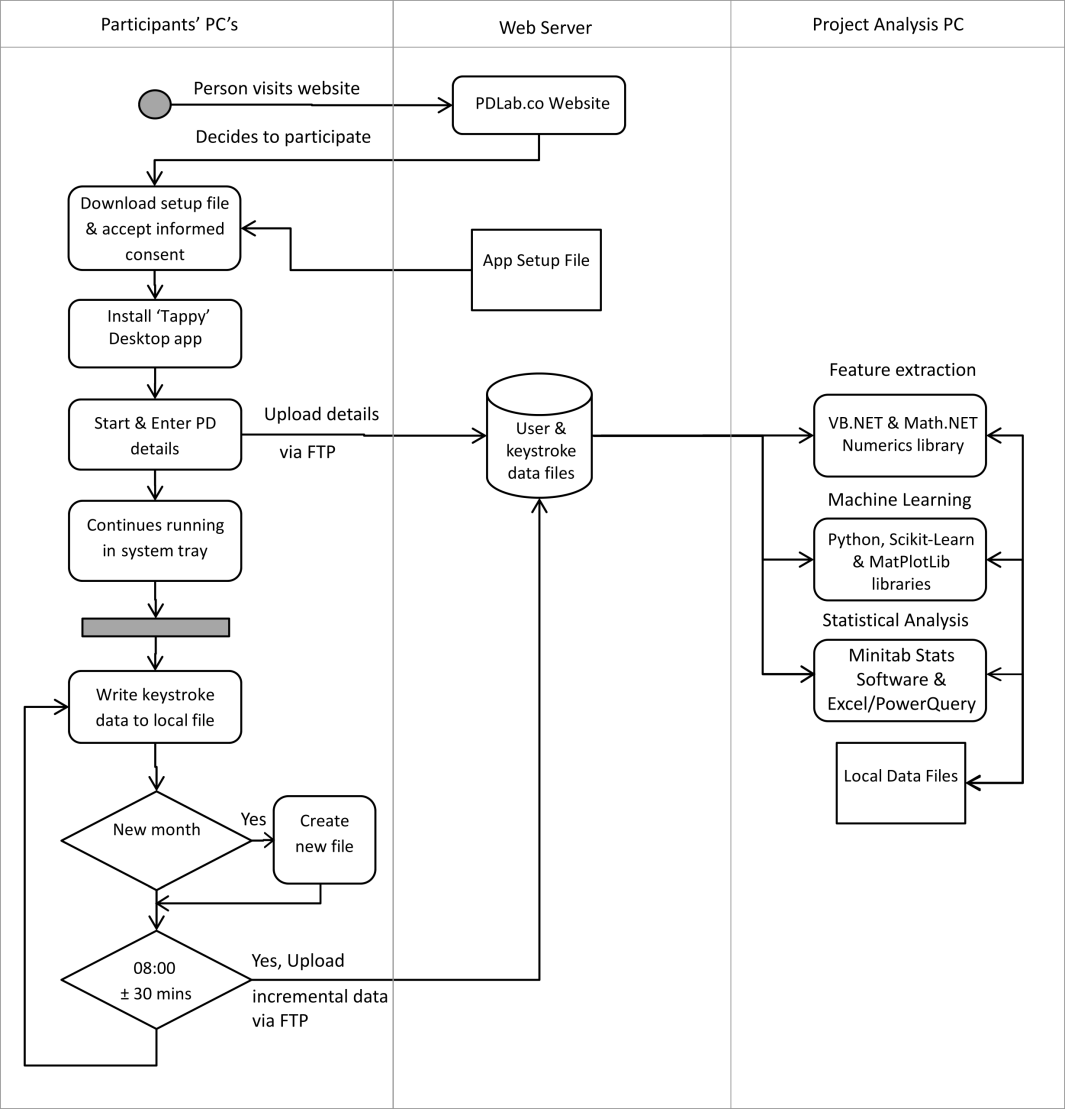
Data collection and processing flow.

The overall data flow for the project is shown in Fig 2, where the custom Tappy application ran as a background process on each participant’s PC and enabled system-wide real-time recording of keystroke data.

**Fig 3:**
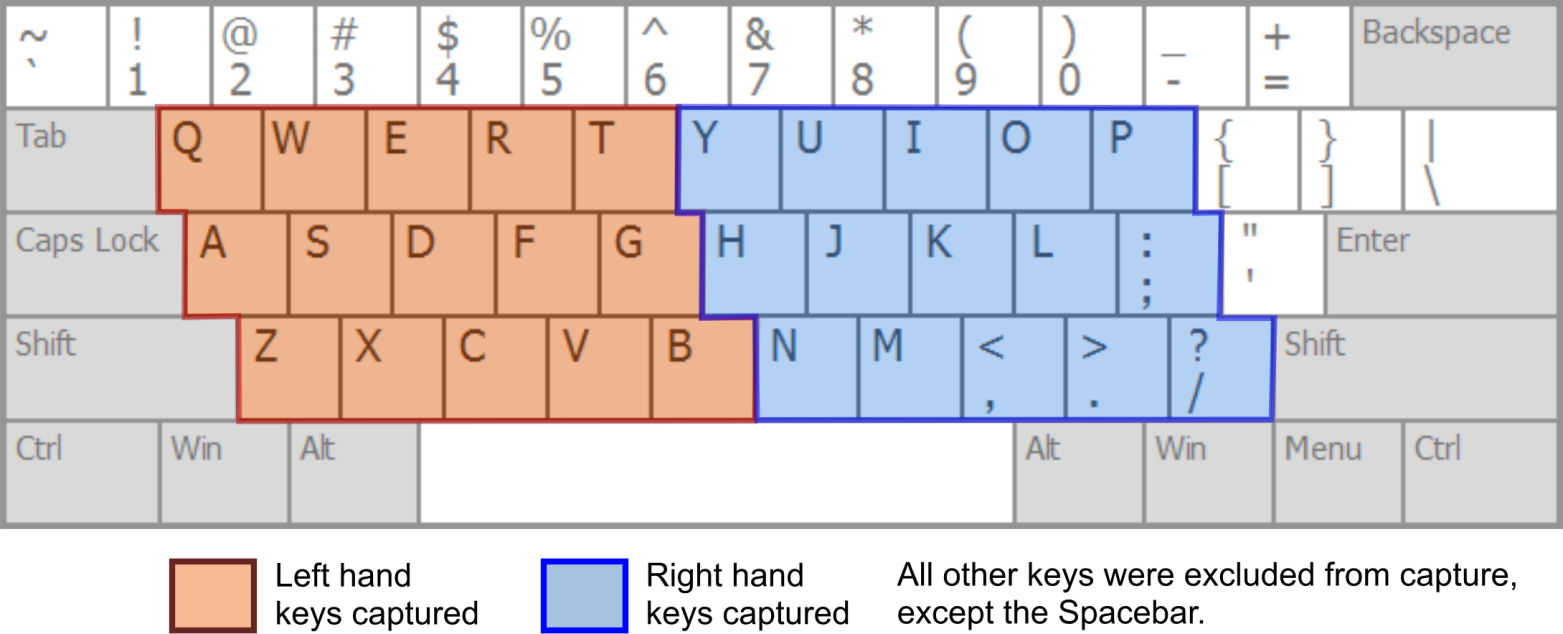
The areas of the keyboard included in keystroke capture.

Not all keystrokes were recorded, just those in the keyboard area corresponding to the five columns for left-hand fingers and the five for right-hand fingers (Fig 3), but excluding all numeric keys. The ‘Tappy’ software utilised the Windows *SetWindowsHookEx* API function to globally hook the keyboard timing events. This approach resulted in a keystroke timing accuracy to within 3 ms [25]. The individual keystroke timings were saved continuously to a CSV file on the participant computer, then incrementally uploaded to a web server once a day via FTP.

An essential consideration in capturing the keystroke data from the participants’ PC’s was to protect the confidentiality of user information. In order to achieve this, participants were completely anonymous, no personal details were recorded and their data was anonymised by using a randomly generated, 10 character identifier. Tappy did not record the actual characters typed, nor record any numeric keys at all, merely classifying whether each key was left or right-hand and the column position on the keyboard. For example, each of the keys E, D and C were identified as simply ‘Left, Column 3’. These steps resulted in the dataset comprising non-identifiable data [27].

### Data analysis

The complete dataset comprised 217 participants (termed ‘Group A’), however only some of those were included the subsequent analysis, comprising

- Those with at least 2000 keystrokes
- Of the ones with PD, just the ones with ‘Mild’ severity (since the study was into the detection of PD at its early stage, not later stages)
- Those not taking levodopa (Sinemet^®^ and the like), in order to prevent any effect of that medication on their keystroke characteristics.

**Fig 4:**
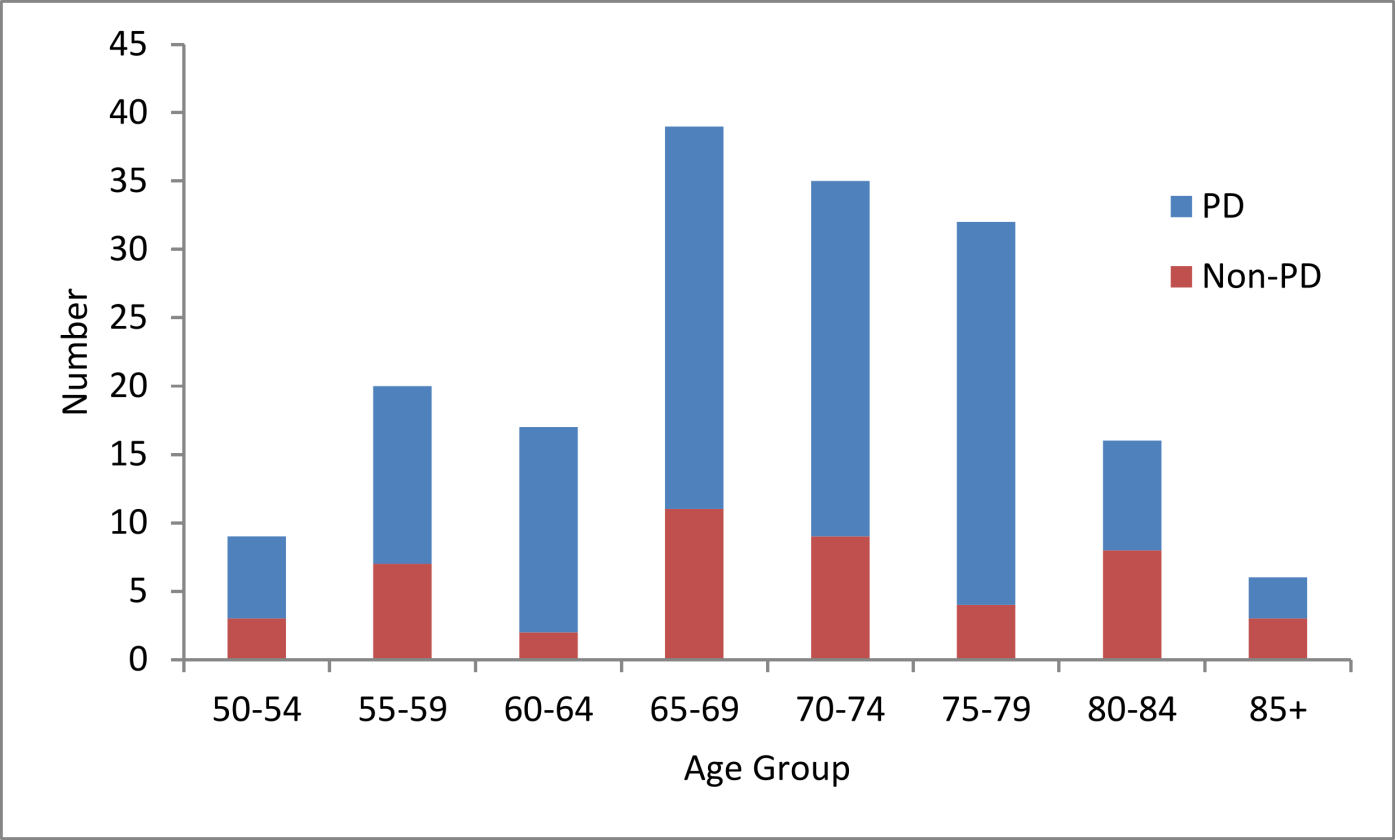
Distribution of participant ages (all participants, both PD and controls).

This produced an analysis subset of 53 participants (including both PD and non-PD), with the characteristics shown in Table 3 and the range of ages in Fig 4.

**Table 3:** Overall characteristics of Tappy participant cohort, Group A.

Characteristic		All Participants		Analysis Subset	
		Count	Percent	Count	Percent
Disease Status	PD	162	75%	20	38%
	Non PD	55	25%	33	62%
	Total	217		53	100%
Gender	Male	116	53%	26	51%
	Female	101	47%	27	49%
PD Tremors	Yes	97	60%	10	50%
	No	65	40%	10	50%
Severity	Mild	68	42%	20	100%
	Medium	70	43%		
	Severe	24	15%		
Sidedness	Left	49	30%	7	35%
	Right	60	37%	8	40%
	None	53	33%	5	25%

There were a total of 27 features extracted from the raw keystroke data (Table 4) by a custom VB.NET application combined with the Math.NET numerics library [28]. Those particular features which were chosen were based on the known movement characteristics of people with PD, as discussed earlier in this report, and included means, standard deviations, skewness and kurtosis. For example, variability in finger movement would be evidenced by variance and skew, jerkiness and hesitation by kurtosis, and so on. The features were then categorised into two groups, one relating to finger movement in the vertical plane (‘Hold’) and the other relating to keystroke n-tuple sequences (‘Latency’), for the subsequent machine learning phase. Features were not necessarily independent—there was some covariance between them and there was also redundant data.

### Machine learning

#### Strategy

In this study, rather than considering the keystrokes as a time series of events, they were treated as a sequence of ordered pairs (n-tuples)–

$$\left(a_{1},a_{2},a_{3},a_{4,}a_{5,}\ldots \;a_{n})\equiv ((a_{1},a_{2}),(a_{2},a_{3}),(a_{3},a_{4}),\ldots \;(a_{n-1},a_{n})\right)$$ (1)

where *a*_*i*_ are the sequence of individual keystrokes.

This approach eliminated the need to have participants type continuous text for a particular duration. Machine learning (ML) models were applied to the analysis dataset, with the goal of achieving the maximum accuracy in correctly classifying each participant as either having, or not having, PD. As well as maximising the area under the curve (AUC) [29], the specificity (minimum number of false positives) was also an essential criteria.

Preliminary analysis had identified that individual features were not a reliable indicator, so the ML strategy focussed on using the combination of multiple features for the classification, rather than analysing individual features. An ensemble method comprising multiple machine learning models was developed (using Python along with the Scikit-Learn library [30], the goal being to combine them into a meta-classifier with a better generalization performance than each individual classifier alone [31,32].

#### Pre-processing

The data was first prepared by creating an *n* by *m* array (with *n* being the number of subjects and *m* the number of features), fitting any missing values (using mean imputation) and normalising the data so that all values were within the range of 0 to 1. The relatively large number of features involved (27 features) compared to the dataset size (53 samples), meant that ‘the curse of dimensionality’ was likely [33], with the potential result of overfitting the training data. This was addressed by the separation the features into two groups, ‘Hold’ (with 9 features) and ‘Latency’ (with 18 features), then applying a dimensional reduction technique, linear discriminant analysis (LDA) [34,35], to each.

Another area where ML is prone to overfitting is leakage between the training and test data, where the model captures the patterns in the training data well, but fails to generalise well to unseen data [36]. This risk was minimised by randomly separating the data into ‘training’ and ‘test’ datasets, along with k-fold cross validation (using a k value of 10) to ensure that all data was represented.

#### Classification

An ensemble of 8 different models was used, comprising a range of classifier types, as shown in Table 5. Each individual model was chosen based on tests of its classification performance on the participant datasets. The justification for using a large ensemble was that, even though there was a significant difference between models in their classification accuracy of individual participants, the accuracy increased as the models were combined with others into a meta-classifier.

**Fig 5:**
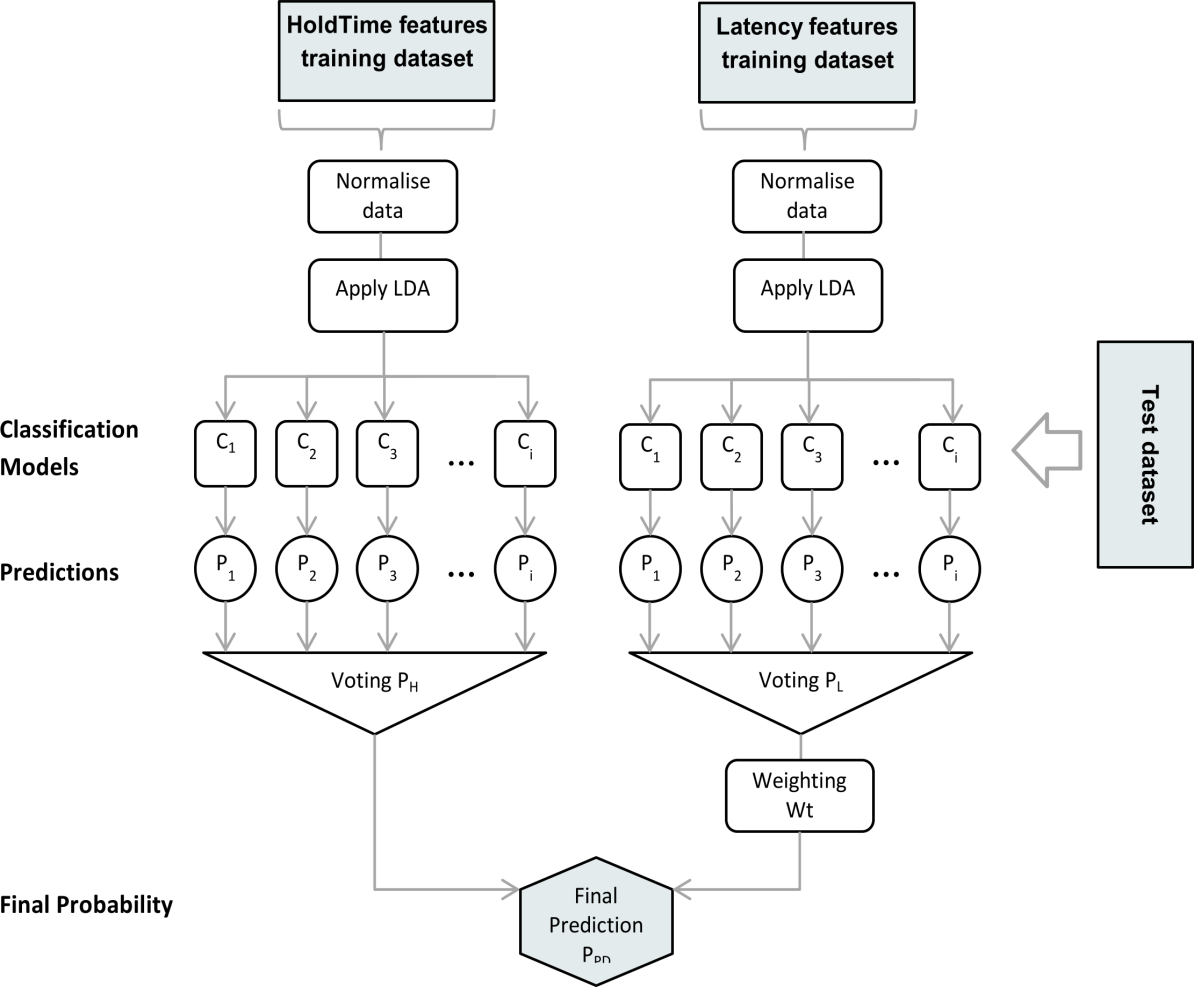
Machine learning flow.

The machine learning flow is shown in Fig 5. Each model was firstly applied to both the Hold and Latency feature groups to generate a set of prediction classifications (PD or non-PD) and predicted probability values for each data sample.

Then, for each of the Hold and Latency groups, the predicted probability results of all the models were averaged (a ‘mean probabilities classifier’), and the final classification step combined those results by weighting the predicted probabilities of each group according to their respective classification accuracy. The predicted classifications for the Latency feature group had a higher overall accuracy than that for the Hold group, so the probabilities were combined as a series of weighted means–

$$P_{PD}=(\sum_{i=1}^{n}P_{{MH}_{i}}+\sum_{i=1}^{n}(0.5+Wt.(P_{{ML}_{i}}-0.5)))/2n$$ (2)

where *P*_*PD*_ is the probability of Parkinson’s for a particular subject (with *P*_*PD*_ ≥ 0.5 classified as having the disease). *MH*_*i*_ and *ML*_*i*_ are the classification models for the Hold and Latency feature groups respectively (Table 4), *P* is the calculated probability for each model, n is the number of ensemble models (8) and *Wt* = 1.2 is a weighting factor for the latency feature group.

**Table 4:** The keystroke features that were extracted for analysis.

Feature Group	Feature	Comments
Hold	Hold time (left fingers)	Hold time is the elapsed time between pressing and releasing each key (that is, movement in the vertical plane).
	Hold time (right fingers)	
	*Each of the above has mean*, *standard deviation*, *skewness & kurtosis*	
	Mean difference between left and right	This is a measure of asymmetry
Latency	Left to right key latency	Latency is the elapsed time between the ordered pairs (n-tuples) of pressing one key and pressing the next key (generally different fingers and often involving fingers of opposite hands).
	Right to left key latency	
	Left to left key latency	
	Right to right key latency	
	*Each of the above has mean*, *standard deviation*, *skewness & kurtosis*	
	Mean difference between LR and RL Mean difference between LL and RR	These are measures of asymmetry,

**Table 5:** Machine learning models used in the classification ensemble.

Model	References and comments
1. Support vector machine (SVM)	[37]
2. Multi Level Perceptron (MLP)	Sensitive to feature scaling, so data normalisation was needed [38]
3. Logistic Regression Model (LRM)	[39]
4. Random forest (RFC)	[40]
5. Nu-Support Vector Classification (NSVC)	A refinement of SVC, with control of the number of support vectors and training errors
6. Decision tree classifier (DTC)	[41]
7. K-nearest neighbours (KNN)	Memorises rather than learns a discriminative function. [42]
8. Quadratic discriminant analysis (QDA)	[43]

#### Data reliability and cross-validation

Only those participants with at least 2000 keystrokes were included, which provided a high degree of confidence in the precision of the feature values, including skewness and kurtosis (which are prone to high variability at smaller sample sizes) and the resulting dataset, termed Group A, totalled 53 participants. The data was split into training and test datasets in a 0.65:0.35 ratio, however this reduced the size of both portions and potentially impacted the classification accuracy. The solution, as previously mentioned, was to implement a cross-validation (CV) procedure, using k-fold cross validation [36]. The advantage of this method was that all samples could be used for both training and validation, and results from the ten k-folds were then averaged to produce a single estimation.

The results of the ML classifications were evaluated on the criteria of maximum sensitivity (true predictions of PD), maximum specificity (minimum false positives) and maximum area under the curve (AUC). Extensive analysis and testing of the performance of the ensemble classifier was also undertaken by–

- Using different voting approaches (in particular, by comparing the performance of the ‘mean probabilities classifier’ to a ‘majority classifier’)
- Excluding particular features (either individually or in combination) and reducing the number of features included in the Hold and Latency groups
- Reducing and/or changing the particular classification models
- Varying the pre-processing (e.g. PCA, LDA and others), and also the techniques used (e.g. bootstrapping)
- Varying the hyper parameters for each model
- Varying the weighting value, Wt in Eq 2.

The outcome of the testing was that optimum results were achieved by including all 8 ML models in both the ‘Hold’ and ‘Latency’ meta-classifiers, and using a weighting value of 1.2 for ‘Latency’. There was a significant improvement in accuracy by including LDA as a pre-processing step. All 27 features were retained and the default parameters for each Scikit-Learn model were used.

### Results validation using an independent dataset

In addition to using the Group A participant data for training and prediction, the ML technique was also applied to a completely independent public dataset (Group B)–the ‘neuroQWERTY MIT-CSXPD’ dataset [44], which was developed in a study by Giancardo et al. [26] and contained keystroke data from 85 subjects as shown in Table 6. A subset of this Group B data, comprising 50 participants (those with just Mild severity, not taking Levodopa, and with at least 500 keystrokes) was used to validate the accuracy of the ML predictions.

**Table 6:** Details of the Group B validation dataset.

Characteristic	Details	Comments
Participants	85 subjects total, mild and medium PD severity, plus controls	
Keystroke sample size	Approximately 1500 and 3000 keystrokes	This included numeric, navigation & punctuation keys. Once all these were removed, the total keystroke sizes were at the lower end of that required for statistical accuracy of the features
Timings	Timestamp, press time, release time, hold time, key	These were able to be converted to equivalent Tappy data elements & timings
Parkinson’s Disease	Validated by a specialist	This ensured the accuracy of the disease classification
Age, gender	Not available	
Diagnosis year	Not available	
LDopa medication		Those participants on LDopa had not taken it for 18 hours prior to the test
Tremors	Not available	This prevented the dataset being used for tremor classification
Sidedness	Not available	This prevented the dataset being used for sidedness classification
UPDRS-III score	Validated by movement disorder specialists	Mild severity is UPDRS < 20
Typing speed		Not relevant
Finger tapping test score	Both single finger (STap) & alternating finger (AFTap)	These scores were from separate finger tapping tests, not from typing

## Results and discussion

### Group A Participants

**Fig 6:**
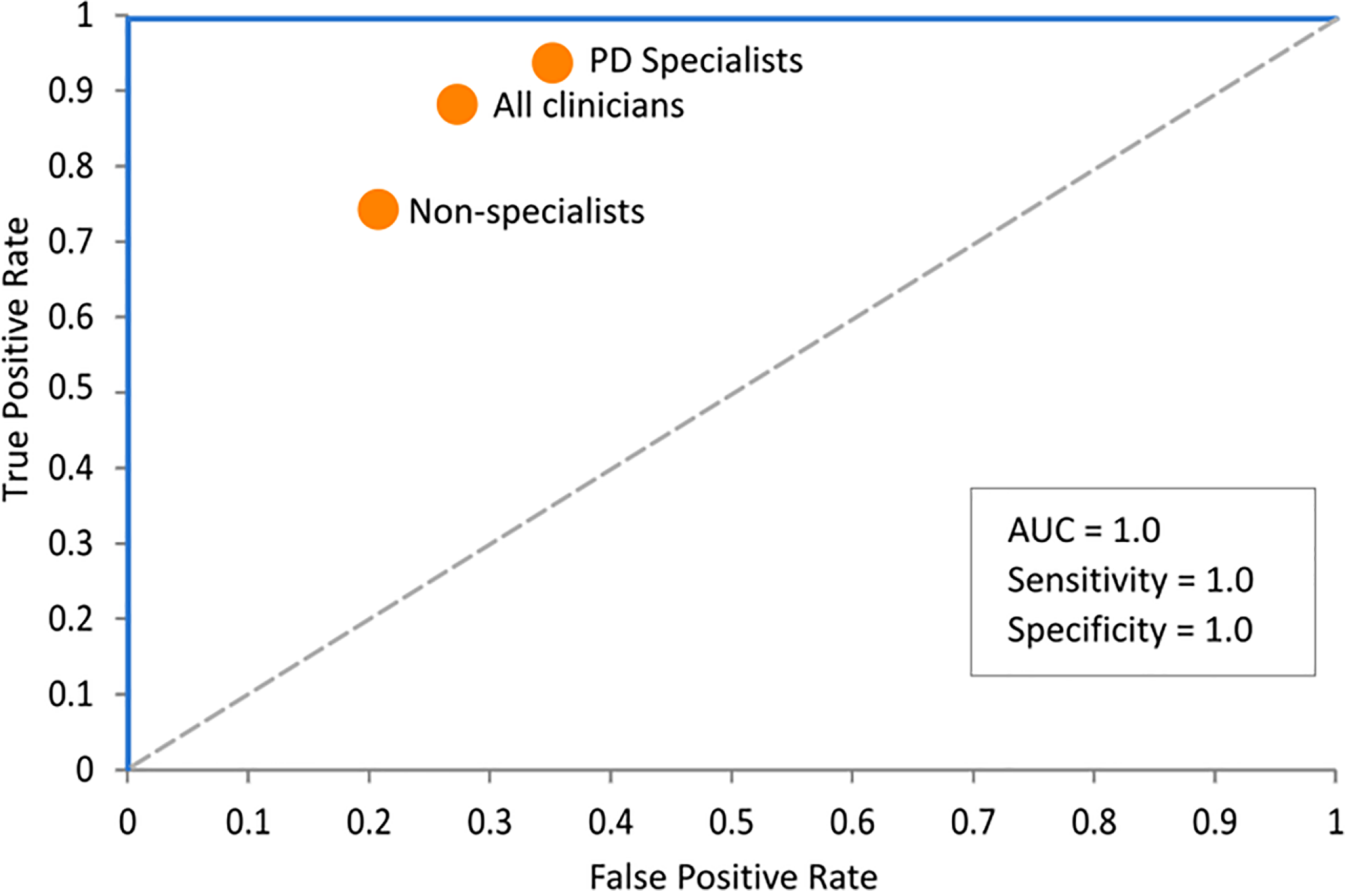
Area under the curve (AUC) for Group A results. (The clinician results shown are from Schrag et al. [45]).

The machine learning ensemble was run on the Group A datasets (using 65% of the data for training and 35% for testing) and achieved a classification accuracy of 100% compared to the participants’ true disease status (Table 7), and an area under the curve (AUC) of 100% (Fig 6).

**Table 7:** Group A machine learning classification, PD and non-PD.

PD / Non-PD	20 / 33
True positives	20
True negatives	33
False positives	0
False negatives (missed detection)	0
Sensitivity = TP / (TP + MP) x 100	100%
Specificity = (1 –(FP / (FP + TN))) x 100	100%
Accuracy = ((TP + TN)/Total) x 100	100%

**Fig 7:**
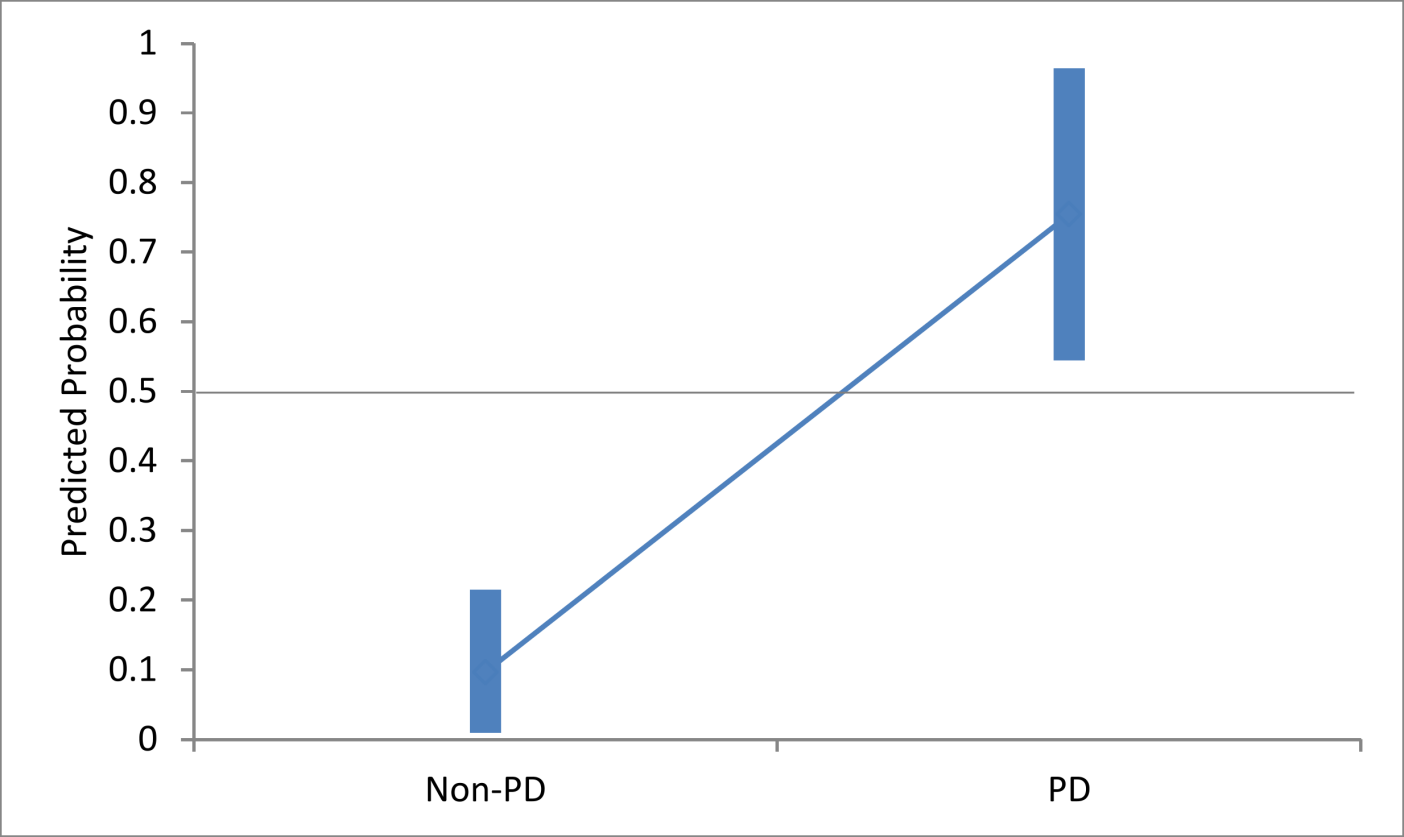
The range of Group A classification probability values. PD and non-PD, showing the 99% confidence interval for each.

A one-way ANOVA was conducted to compare the probability values of those predicted as having, or not having, PD. There was a significant difference between them at the p < 0.01 level [Welch’s F(1, 12.224) = 86.71, p < 0.001], indicating a high ability of the ML classification technique, even with a limited sample size (Fig 7).

### Group B Participants

**Fig 8:**
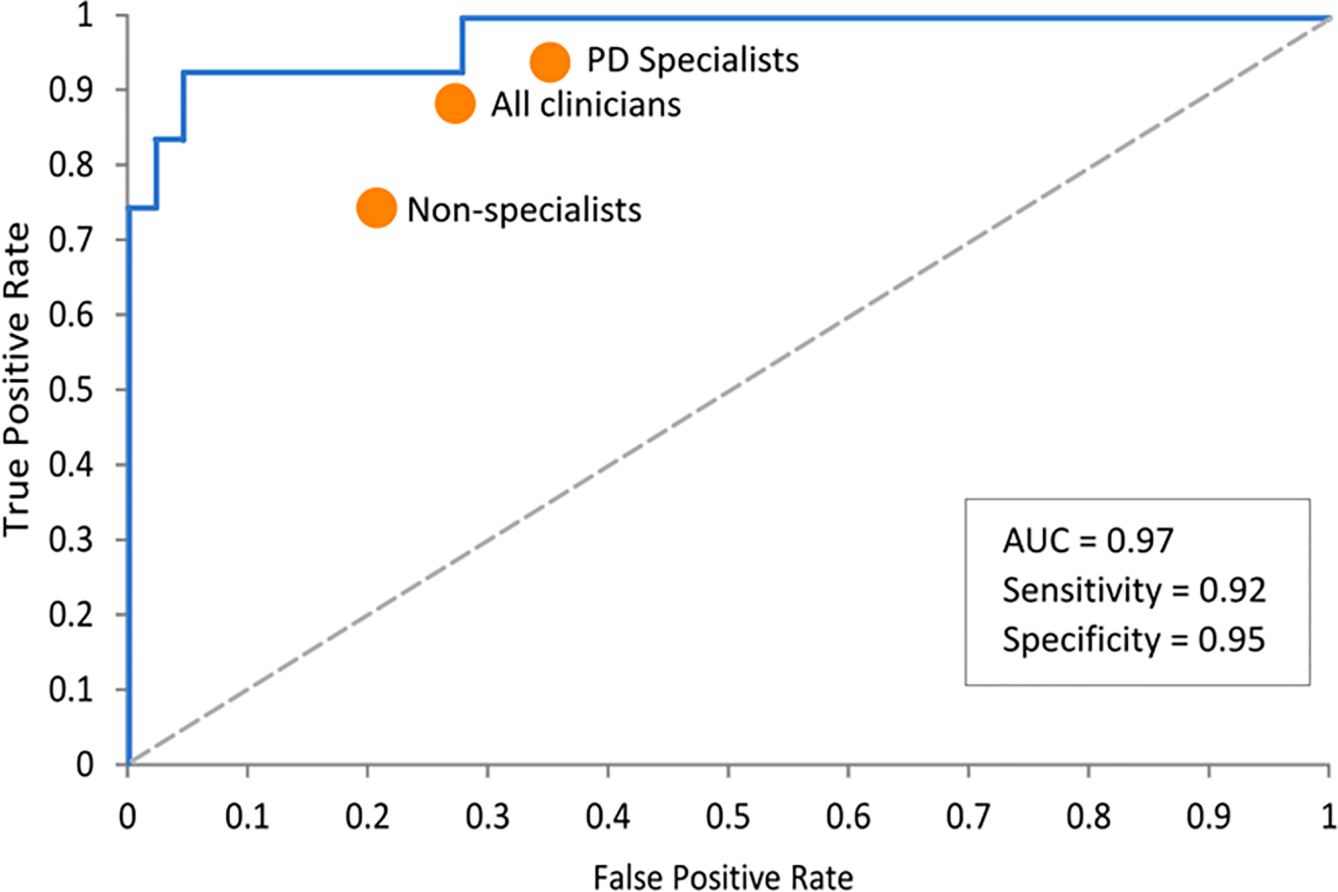
Area under the curve (AUC) for Group B results.

The predicted classifications for the independent dataset, Group B, were also calculated, in this case using the entire Group A data as the training set. This achieved an accuracy of 94% compared to the participants’ true disease status (Table 8) and an AUC of 97% (Fig 8).

**Table 8:** Group B machine learning classification, PD and non-PD.

PD / Non-PD	12 / 38
True positives	11
True negatives	36
False positives	2
False negatives (missed detection)	1
Sensitivity = TP / (TP + MP) x 100	92%
Specificity = (1 –(FP / (FP + TN))) x 100	95%
Accuracy = ((TP + TN)/Total) x 100	94%

**Fig 9:**
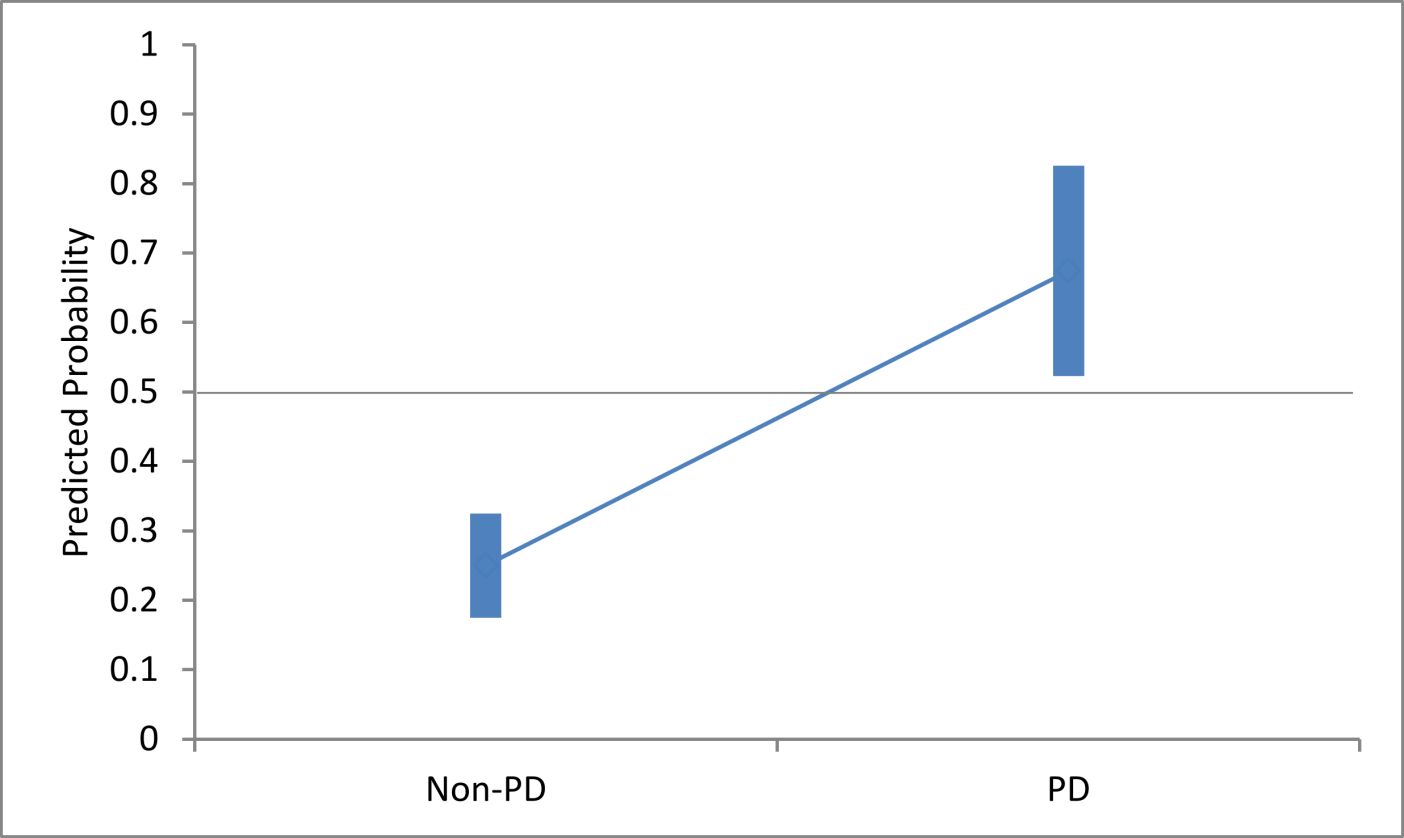
The range of Group B classification probability values. PD and non-PD, showing the 99% confidence interval for each.

A one-way ANOVA showed a significant difference between the classification probabilities at the p < 0.01 level [Welch’s F(1, 18.465) = 57.80, p < 0.001], indicating a high ability of the ML classifier to differentiate between those with PD and those without (Fig 9).

A reason for the differences in the detection accuracy of Group B compared to Group A is likely to be simply because of the much smaller number of keystrokes for the Group B subjects (some with only 500 keystrokes), in contrast to Group A subjects (who all had at least 2000 keystrokes). Despite this limitation, the Group B results still exceeded those of clinicians and demonstrated the applicability of the ML ensemble.

### Comparison of detection accuracy with existing diagnostic techniques

Using data from Schrag et al. [45], Figs 6 and 8 also show the equivalent diagnostic accuracy (sensitivity and specificity) achieved by clinicians, which demonstrates that the ML results not only significantly outperformed non-specialist clinicians, but had a much lower false positive rate compared to PD specialists, (with the details shown in Table 9).

**Table 9:** Diagnosis accuracies achieved in this research, compared to clinicians and other techniques.

Diagnosis Type	Sensitivity	Specificity	AUC
Tappy (this investigation)	92% to 100%	95% to 100%	97% to 100%
Non-specialist clinicians	74%	79%	-
Specialists (neurologists & movement specialists)	93%	65%	-
Smartphone accelerometer—forearm pronation & supination (FPSMT), [46]	86%	89%	-
NeuroQWERTY study [26]	71%	84%	81%
Alternating finger tap test (AFTT)	72% (est)	69% (est)	75%
Single finger tap test (SFTT)	59% (est)	58% (est)	61%

In comparison to other HCI techniques which have been developed recently, for example, smartphone forearm pronation/supination tests, the ML technique of this research not only gave superior results, but was also the only technique which did not require supervision.

### Application and future research

This research has been the first HCI technique to achieve a diagnostic accuracy significantly greater than that of non-specialist clinicians. It was also more accurate than any existing quantifiable test and was able to detect PD in its early stages, specifically where there were just mild symptoms present, and where the typical characteristics of PD (for example, tremors and sidedness) were not necessarily evident.

The techniques utilised in this investigation resulted in a combined meta-classifier model that could be saved and applied directly to new data, which means that it could potentially form the basis of a diagnostic suite of HCI tools for Parkinson’s Disease, for use by family practice physicians and general practitioners.

There are several aspects of the study for which further investigation is intended, in particular to increase the participant numbers (‘training set’ size) in order to further enhance the reliability of the technique. Subsequent investigations will also investigate the detection of tremors in PD patients (as a second cardinal symptom), the relative importance of the keystroke features used and, through a longitudinal study, the manner in which features change over time as the disease progresses.

## Conclusion

In this investigation, keystroke timing information from 103 subjects, including 32 with just mild PD severity, was captured as they typed on a computer keyboard over an extended period. It showed that PD does affect various characteristics of hand and finger movement, which can be detected from keystroke features. A novel methodology was used to classify the subjects’ disease status, by utilising a combination of extracted features from those keystrokes which were then analysed by an ensemble of machine learning classification models.

This approach was able to discriminate between early-PD subjects and controls with a sensitivity of 92 to 100%, a specificity of 95 to 100%, and an AUC of between 0.97 and 1.00. These results are significantly more accurate than that achieved by previous HCI studies and it is the first technique to exceed the diagnostic accuracy of non-specialist clinicians, which suggests that it can provide an objective, accurate detection of PD, especially in its early stages where motor symptoms such as tremors or sidedness may not yet be observable. At present the technique does not incorporate a second cardinal PD motor symptom (such as tremors or rigidity for example) so, by itself, cannot differentiate between PD and other movement-related conditions.

Less than 400 words of typing is needed for reliable detection, the technique does not require any specialised equipment or attachments, does not need medical supervision, does not rely on the experience and skill of the practitioner, and can take place in the patient’s home or office environment as they type normally on a computer.
